# Incidence of postoperative complications in transabdominal preperitoneal repair for groin hernia is influenced by poor performance status rather than by old age

**DOI:** 10.1002/ags3.12247

**Published:** 2019-04-04

**Authors:** Noriyuki Egawa, Jun Nakamura, Tatsuya Manabe, Hironori Iwasaki, Hirokazu Noshiro

**Affiliations:** ^1^ Department of Surgery Faculty of Medicine Saga University Saga Japan

**Keywords:** elderly, groin hernia, transabdominal preperitoneal

## Abstract

**Aim:**

The present study was designed to evaluate the safety and feasibility of transabdominal preperitoneal (TAPP) repair for very old patients with groin hernia and to identify the risk factors predicting perioperative complications.

**Methods:**

A total of 140 patients treated by TAPP were reviewed retrospectively. They were divided into two groups: patients ≥80 years of age (≥80 years group; n = 26) and those <80 years of age (<80 years group; n = 114). Patient characteristics and surgical outcomes were then statistically compared between the two groups.

**Results:**

Number of patients with any comorbidities was significantly higher in the ≥80 years group than in the <80 years group (96.2% vs 61.4%, *P* = 0.003). There were no significant differences in surgical outcomes between the two groups. In the univariate analysis of perioperative complications, poor performance status (PS) (*P* = 0.014), lower hemoglobin level (*P* = 0.038) and lower albumin level (*P* = 0.016) were significantly associated with the occurrence of postoperative complications, and multivariate analysis showed that only poor PS was an independent factor (PS 0‐2 vs 3‐4: *P* = 0.034, OR 5.192 [95% CI; 1.137 to 23.71]).

**Conclusions:**

This is the first report to show that the incidence of postoperative complications in TAPP repair for groin hernia is influenced by poor PS rather than old age. TAPP can be a safe surgical procedure for very old patients with a good PS, with benefits that are equal to those in young patients.

## 1 INTRODUCTION

Laparoscopic repair of groin hernia by the totally extraperitoneal (TEP) or transabdominal preperitoneal (TAPP) approach is an established surgical method for treating groin hernia and has been carried out widely in recent years.^1^, ^2^ Previous studies have reported that laparoscopic hernia surgery (LHS) results in a lower incidence of wound infection, chronic pain and numbness and an earlier return to work than open hernia surgery (OHS).^3^, ^4^, ^5^ However, LHS requires general anesthesia and a longer operation time than OHS. In addition, LHS has a higher incidence of postoperative complications than OHS, and TAPP, in particular, seems to be associated with serious complications, such as port‐site hernia and visceral injury.^3^, ^6^, ^7^ Based on these findings, current guidelines recommend LHS as an option for the treatment of primary unilateral groin hernia when carried out by a sufficiently experienced surgeon.^8^, ^9^

Because the incidence of groin hernia is higher in the elderly than in younger individuals due to the weakening of tissue with age,^1^, ^10^, ^11^, ^12^ the number of groin hernia surgeries for elderly patients is expected to increase as the population progressively ages in developed countries. Furthermore, postoperative morbidity and mortality rates in major surgery are also higher in elderly patients than in young patients.^13^, ^14^, ^15^ Elderly patients often receive many medications and have multiple comorbidities and histories of laparotomy that can hamper laparoscopic surgery. Therefore, older age may be a risk factor predicting postoperative complications in LHS. Several studies have reported comparable outcomes between LHS and OHS in elderly patients;^16^, ^17^, ^18^ however, whether older age influences the incidence of postoperative complications in LHS has been unclear. Therefore, objective parameters regarding the selection of laparoscopic surgery for elderly groin hernia patients are needed.

The purpose of the present study was to evaluate the safety and feasibility of TAPP repair for very old patients with groin hernia and to identify the risk factors predicting postoperative complications in this population.

## 2 PATIENTS AND METHODS

**Figure 1:**
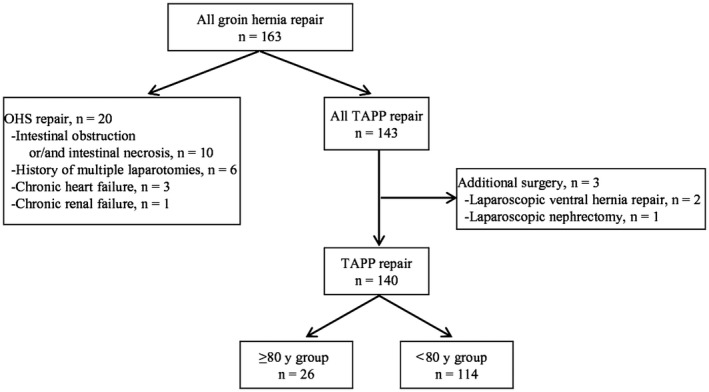
Study flow chart. OHS, open hernia surgery; TAPP, transabdominal preperitoneal

From September 2011 to May 2016, 163 patients underwent groin hernia repair at Saga University Hospital. During this period, almost all groin hernia patients were treated by TAPP repair when the patient's condition allowed for laparoscopic surgery under general anesthesia. Among 163 groin hernia patients, 20 were treated by OHS, whereas the remaining 143 were treated by TAPP. Selection criteria for OHS were as follows: contraindication for general anesthesia as a result of severe comorbidities (n = 4); severe intra‐abdominal adhesion as a result of multiple laparotomies (n = 6); and emergency cases as a result of intestinal obstruction and/or necrosis (n = 10). Furthermore, among 143 patients who underwent TAPP, three who received additional surgery at the same time were excepted. Therefore, this study retrospectively reviewed 140 consecutive groin hernia patients who received TAPP. These 140 patients were divided into two groups: patients ≥80 years of age (≥80 years group; n = 26) and those <80 years of age (<80 years group; n = 114) (Figure 1). Patient characteristics and surgical outcomes including operation time, blood loss, hospital stay and postoperative complications were statistically compared between the two groups. Details of comorbidity are as follows: pulmonary dysfunction was defined as % vital capacity (%VC) ≤80% or forced expiratory volume in 1‐second (FEV1.0%) ≤70%; renal dysfunction was defined as estimated glomerular filtration rate (eGFR) ≤60 mL/min. Circulatory disease included myocardial infarction, arrhythmia and valvular heart disease. Postoperative complications were classified according to the Clavien‐Dindo classification of surgical complications,^19^ and complications classified as grade 2 or higher were reviewed.

### 2.1 Statistical analyses

Data analyses were carried out using JMP Pro 13 (SAS Institute Inc., Cary, NC, USA) statistical software. All data are expressed as mean ± standard deviation. Fisher's exact test or a two‐tailed Student's *t*‐test was used to evaluate group differences. Variables with *P* values ≤0.05 on univariate analysis were included in a multivariate logistic regression model. All *P* values <0.05 were accepted as statistically significant.

## 3 RESULTS

### 3.1 Patient characteristics

Table 1 compares the characteristics between the ≥80 years group (n = 26) and the <80 years group (n = 114) among patients who received TAPP. Mean age was 83.6 ± 3.1 years old in the ≥80 years group and 64.3 ± 13.4 years old in the <80 years group (*P* < 0.001). Body mass index (BMI), American Society of Anesthesiologists (ASA) class, performance status (PS), type of groin hernia and size of hernia orifice were similar between the two groups. Lower hemoglobin (Hb) (12.8 ± 1.6 mg/dL vs 13.7 ± 2.7 mg/dL, *P* = 0.038) and albumin (Alb) levels (3.7 ± 0.4 mg/dL vs 4.0 ± 0.5 mg/dL, *P* = 0.016) were observed in the ≥80 years group. No significant differences regarding the proportions of emergency operations, recurrent cases and the histories of laparotomy, prostatectomy, antithrombotic therapy and steroid therapy were observed between the two groups. The proportion of patients with any comorbidities (96.2% vs 61.4%, *P* = 0.003), especially those having renal dysfunction (57.8% vs 20.2%, *P* = 0.003), pulmonary dysfunction (%VC ≤80% or FEV1.0% ≤70%) (53.9% vs 15.8%, *P* < 0.001), and cerebrovascular infarction (23.1% vs 3.5%, *P* = 0.003) was significantly higher in the ≥80 years group than in the <80 years group. It is noteworthy that over 95% of the patients in the ≥80 years group had some comorbidities. The proportion of patients with comorbidities of circulatory disease, liver disease, diabetes, and dementia did not differ to a statistically significant extent.

**Table 1:** Characteristics of TAPP repair patients

	≥80 y group (n = 26)	<80 y group (n = 114)	*P* value
Gender, male/female	22/4	98/16	0.766
Age (y, mean ± SD)	83.6 ± 3.1	64.3 ± 13.4	<0.001
BMI (kg/m^2^, mean ± SD)	21.6 ± 2.7	22.5 ± 2.7	0.101
Performance status (0‐2/3‐4)	23/3	109/5	0.167
ASA classification (1‐2/3‐4)	25/1	111/3	0.565
Blood test			
Hb (mg/dL, mean ± SD)	12.8 ± 1.6	13.7 ± 2.7	0.038
Alb (mg/dL, mean ± SD)	3.7 ± 0.4	4.0 ± 0.5	0.016
Diseased side (unilateral/bilateral)	25/1	100/14	0.304
Type of groin hernia^a^ (lateral/medial/femoral/combined)	21/5/1/0	93/28/2/5	0.635
Size of hernia orifice^b^ (<3 cm/≥3 cm)	20/6	98/16	0.392
Emergency operation, n (%)	2 (7.7)	2 (1.8)	0.157
Recurrent case, n (%)	3 (11.5)	11 (9.7)	0.724
History of other side inguinal hernia repair^c^, n (%)	3 (12.0)	17 (17.0)	0.762
History of abdominal surgery, n (%)	14 (53.9)	41 (36.0)	0.12
History of prostatectomy^d^, n (%)	2 (9.1)	9 (9.2)	1.000
Antithrombotic therapy, n (%)	9 (34.6)	27 (23.7)	0.319
Steroid therapy, n (%)	0 (0.0)	4 (3.5)	1.000
Comorbidity, n (%)	25 (96.2)	70 (61.4)	0.003
Circulatory disease, n (%)	9 (34.6)	26 (22.8)	0.218
Renal dysfunction, n (%)	15 (57.7)	23 (20.2)	0.003
Pulmonary dysfunction, n (%)	14 (53.9)	18 (15.8)	<0.001
Liver disease, n (%)	2 (7.7)	12 (10.5)	1.000
Cerebral infarction, n (%)	6 (23.1)	4 (3.5)	0.003
Diabetes, n (%)	7 (26.9)	18 (15.8)	0.254
Dementia, n (%)	2 (7.7)	2 (1.8)	0.157
Surgeon (resident/expert^e^)	17/9	64/50	0.385

Alb, albumin; ASA, American Society of Anesthesiologists; BMI, body mass index; Hb, hemoglobin; PS, performance status; TAPP, transabdominal preperitoneal.

a Including bilateral cases. Described according to European Hernia Society classification.

b In bilateral cases, the larger size of defect was listed. Board Certified Surgeon in Gastroenterology.

c Excluding bilateral cases.

d Excluding female cases.

e Board‐certified surgeon in gastroenterology of the Japanese Society of Gastroenterological Surgery.

### 3.2 Surgical outcomes and perioperative complications of TAPP

Surgical outcomes and perioperative complications of TAPP repair were compared between the ≥80 and <80 years groups (Table 2). There were no significant differences in the respective surgical outcomes between the ≥80 and <80 years groups, including operation time (125.8 ± 40.4 minutes vs 137.5 ± 45.7 minutes, *P* = 0.230), rate of conversion to open surgery (0% vs 0.9%, *P* = 1.000), blood loss (0.9 ± 2.1 g vs 1.5 ± 4.5 g, *P* = 0.510) and postoperative hospitalization duration (3.8 ± 1.4 days vs 3.3 ± 1.6 days, *P* = 0.140). Of all 140 patients who underwent TAPP, only one case (0.7%) had to be converted to open surgery. This patient was diagnosed with an irreducible inguinal hernia and received TAPP electively; however, the incarcerated omentum was unable to be returned into the abdominal cavity laparoscopically. Although the hernia defect was able to be repaired laparoscopically, an additional incision at the groin was needed to excise the strangulated omentum.

**Table 2:** Surgical outcomes and perioperative complications of TAPP repair patients

	≥80 y group (n = 26)	<80 y group (n = 114)	*P* value
Operation time (min), mean ±SD	125.8 ± 40.4	137.5 ± 45.7	0.230
Conversion to open surgery, n (%)	0 (0)	1 (0.9)	1.000
Blood loss (g), mean ± SD	0.9 ± 2.1	1.5 ± 4.5	0.510
Postoperative hospitalization (d), mean ± SD	3.8 ± 1.4	3.3 ± 1.6	0.140
Perioperative complications^a^, n (%)	9 (34.6)	22 (19.3)	0.116
Intraoperative complications, n (%)	0 (0)	1 (0.9)	1.000
Other organ injury	0 (0)	1 (0.9)	1.000
Postoperative complications, n (%)	9 (34.6)	21 (18.4)	0.109
Seroma	5 (19.2)	19 (16.7)	0.775
Hematoma	3 (11.5)	0 (0)	0.006
Wound infection	0 (0)	0 (0)	–
Pneumonia	0 (0)	1 (0.9)	1.000
Cystitis	0 (0)	1 (0.9)	1.000
Other	1 (3.8)	0 (0)	0.186
Inguinal pain and/or discomfort^b^	0 (0)	7 (6.1)	0.348

TAPP, transabdominal preperitoneal.

a Complications grade 2 or higher according to the Clavien‐Dindo classification.

b Observation at 1 mo after surgery.

Rate of overall perioperative complications was similar between the two groups (34.6% vs 19.3%, *P* = 0.116). In addition, there were no significant differences in the rates of both intraoperative complications (0% vs 0.9%, *P* = 1.000) and postoperative complications (34.6% vs 18.4%, *P* = 0.109). Although the incidence of hematoma was significantly higher in the ≥80 years group (11.5% vs 0%, *P* = 0.006), the incidences of other complications, such as seroma, wound infection and visceral injury, were similar between the two groups. There were no mortalities in either group. Pain and/or discomfort that lasted more than 1 month after surgery was more frequent in the <80 years group, although not to a statistically significant degree (0% vs 6.1%, *P* = 0.348).

### 3.3 Identification of risk factors predicting perioperative complications of TAPP

Univariate and multivariate analyses regarding the perioperative complications of TAPP repair are shown in Table 3. In the univariate analysis, poor PS was significantly associated with the occurrence of postoperative complications (*P* = 0.014). Furthermore, Hb level (12.9 ± 1.9 mg/dL vs 13.7 ± 1.8 mg/dL, *P* = 0.036) and Alb level (3.8 ± 0.4 mg/dL vs 4.0 ± 0.5 mg/dL, *P* = 0.035) were significantly lower in the patients with complications. Perioperative complications also tended to be more frequent among elderly and male patients (≥80 years vs <80 years; *P* = 0.116, male vs female; *P* = 0.076), although not to a statistically significant degree. There were no correlations between the perioperative complications and other factors, such as BMI (*P* = 0.511), ASA class (*P* = 1.000), size of hernia orifice (*P* = 0.419), emergency operation (*P* = 1.000), history of laparotomy (*P* = 0.835), antithrombotic therapy (*P* = 0.646), steroid therapy (*P* = 0.213) and presence of comorbidities (*P* = 0.126). Incidence of perioperative complications in procedures carried out by residents and experts did not differ to a statistically significant extent (*P* = 0.385). In addition, multivariate analysis showed that only poor PS was an independent factor predicting the occurrence of perioperative complications (PS 0‐2 vs 3‐4: *P* = 0.034, odds ratio: 5.192 [95% confidence interval: 1.137‐23.71]).

**Table 3:** Univariate and multivariate analyses of perioperative complications in TAPP repair patients

	Univariate analysis			Multivariate analysis		
	Complications^a^ (+) (n = 31)	Complications (−) (n = 109)	*P* value	OR	95% CI	*P* value
Age (≥80 y/<80 y)	9/22	17/92	0.116			
Gender (male/female)	30/1	90/19	0.076			
BMI (kg/m^2^, mean ± SD)	22.1 ± 2.4	22.4 ± 2.8	0.511			
Performance status (0‐2/3‐4)	26/5	106/3	0.014	5.192	1.137‐23.71	0.034
ASA classification (1‐2/3‐4)	30/1	106/3	1.000			
Blood test						
Hb (mg/dL, mean ± SD)	12.9 ± 1.9	13.7 ± 1.8	0.036	1.130	0.864‐1.479	0.371
Alb (mg/dL, mean ± SD)	3.8 ± 0.4	4.0 ± 0.5	0.035	1.821	0.604‐5.493	0.287
Type of groin hernia^b^ (lateral/medial/femoral/combined)	25/10/0/0	89/23/3/5	0.420			
Diseased side (unilateral/bilateral)	27/4	98/11	0.742			
Size of hernia orifice^c^ (<3 cm/≥3 cm)	24/7	92/17	0.419			
Emergency operation (yes/no)	1/30	3/106	1.000			
Recurrent case (yes/no)	3/28	11/98	1.000			
History of laparotomy (yes/no)	13/18	42/67	0.835			
Antithrombotic therapy (yes/no)	9/22	27/82	0.646			
Steroid therapy (yes/no)	2/29	2/107	0.213			
Comorbidity (yes/no)	25/6	70/39	0.126			
Circulatory disease (yes/no)	9/22	26/83	0.639			
Renal dysfunction (yes/no)	8/23	30/79	1.000			
Pulmonary dysfunction (yes/no)	10/21	22/87	0.224			
Liver disease (yes/no)	4/27	10/99	0.511			
Cerebral infarction (yes/no)	2/29	8/101	1.000			
Diabetes (yes/no)	4/27	21/88	0.596			
Dementia (yes/no)	2/29	2/107	0.213			
Surgeon, (resident/expert^d^)	20/11	61/48	0.392			

Alb, albumin; ASA, American Society of Anesthesiologists; BMI, body mass index; Hb, hemoglobin; TAPP, transabdominal preperitoneal.

a Complications grade 2 or higher according to the Clavien‐Dindo classification.

b Including bilateral cases. Described according to European Hernia Society classification.

c In bilateral cases, the larger size of defect is listed.

d Board‐certified surgeon in gastroenterology of the Japanese Society of Gastroenterological Surgery.

## 4 DISCUSSION

Groin hernia repair is one of the most common surgical procedures carried out worldwide.^19^ According to a population‐based study by Zendejas et al,^20^ the number of patients who undergo inguinal hernia repair increases with age, resulting in a cumulative incidence of 18.9% by age 70, 27.7% by age 80, 35.1% by age 90 and 42.5% for the entire lifetime for men. Currently, individuals over the age of 80 years are not unusual in Japan, where individuals aged over 65 years comprise 27.3% of the population.^21^ Some reports allowed for non‐surgical management (watchful waiting) of asymptomatic or minimally symptomatic groin hernias in elderly patients.^22^, ^23^, ^24^, ^25^ However, it is generally considered that symptomatic groin hernias should be treated by surgery to prevent incarceration and reduce mortality,^8^, ^22^, ^23^ and strangulated hernias should be urgently operated, regardless of the patient's age. However, the current guidelines make no recommendations regarding the optimum age‐specific surgical procedures for groin hernias.

Previous studies have described similar surgical outcomes between LHS and OHS in patients over 80 years of age and concluded that LHS is safe and can be an alternative procedure to OHS even in very elderly patients.^17^, ^18^ However, these two studies included a very small number of patients who received LHS, and the selection criteria for LHS or OHS were not described. Recently, Vigneswaran et al evaluated the surgical outcomes of inguinal hernia repair in detail for each age group.^16^ In their study, 380 of 471 patients were treated by the TEP approach, and 13 of 380 patients who received TEP were older than 80 years. The results suggested that patient‐reported outcomes may be better in LHS than in OHS, regardless of a greater risk of minor postoperative complications such as seroma and urinary retention in elderly patients compared with non‐elderly patients. Mayer et al also showed that the rate of perioperative complications in LHS tends to increase with age, in particular from the age of 80 years.^12^ However, to our knowledge, no studies have compared the outcomes of TAPP repair between elderly and non‐elderly patients in consecutive cases of groin hernia.

In the present study, despite the higher rate of underlying disease in elderly patients, there were no significant differences in the surgical outcomes of TAPP repair between the elderly group and the non‐elderly group. Although postoperative hematoma was more frequently observed in elderly patients, overall morbidity was similar between the two groups as reported in the previous studies. Low Hb and Alb levels were associated with perioperative complications in univariate analyses; however, multivariate analyses showed that only poor PS was an independent risk factor that predicted perioperative complications. Other factors including elderly age were not associated with perioperative complications. These results suggest that TAPP can be one of the optimal treatment options even for very old patients as well as for young patients when the patient's condition allows LHS under general anesthesia and PS is 0 to 2. On the contrary, even for young patients, TAPP may provide less advantage compared with OHS when the patient's PS is poor. This can lead to the occurrence of perioperative complications and delay the return to daily life. Generally, the risk assessment of LHS was based on patient's age, underlying diseases, type of hernia, size of hernia orifice, ASA class etc;^12^, ^16^, ^26^, ^27^ however, no studies investigated the association between patient's PS and the incidence of perioperative complications. This study for the first time showed that poor PS is an independent predictive factor of perioperative complications after TAPP. More careful preoperative assessment is needed to decide the surgical procedure for poor PS patients.

Generally, when TAPP is carried out for patients with groin hernia, general anesthesia and intraperitoneal manipulation become major concerns. In other words, if general anesthesia is possible, then it seems that TAPP can be carried out safely, regardless of the patient's age. However, this study did not conclude that the surgical outcomes of TAPP for elderly patients were superior to those of TAPP for young patients. Furthermore, it did not show that TAPP was superior to OHS for elderly patients with groin hernia. To obtain a consensus regarding the optimal surgical procedure for elderly patients and/or poor PS, comparison between LHS and OHS in such patients will be needed.

In the present study, there were no patients with chronic postoperative inguinal pain requiring long‐term follow up or drug treatment. However, there was a tendency for young people to feel postoperative inguinal pain or discomfort in the first month after surgery, although there was no significant difference. Young age was regarded as a risk factor for postoperative pain in previous reports; however, these reports were mainly based on research on OHS.^28^, ^29^ In reports on LHS, some studies noted that age did not have a significant influence on postoperative inguinal pain;^30^, ^31^ however, this point remains controversial.

The major limitations of the present study are its retrospective design and small sample size. In addition, many surgeons from resident to expert carried out operations in this study. The preoperative complication rate did not differ between procedures carried out by residents and experts; however, experts may have carried out more difficult operations such as hernia after total prostatectomy, recurrent hernia, and hernia of high‐risk patients including the very elderly.

## 5 CONCLUSION

The present study for the first time showed that the incidence of perioperative complications in TAPP repair for groin hernia is influenced by poor PS rather than old age. In conclusion, when the condition of the patient allows general anesthesia and PS is 0‐2, TAPP can be one of the optimal surgical procedures even for very elderly patients over 80 years of age, as it is for young patients. Very elderly patients with a good PS can enjoy the benefits of TAPP equally to young people.

## DISCLOSURE

This study was carried out in agreement with the guidelines of the institutional ethics committee and was conducted in accordance with the Declaration of Helsinki.

Conflicts of Interest: Authors declare no conflicts of interest for this article.
